# Application of Cold Plasma to Reduce the Roughness of the Working Surface of a Railway Car Center Plate

**DOI:** 10.3390/ma17225437

**Published:** 2024-11-07

**Authors:** Maryna Bulakh

**Affiliations:** Faculty of Mechanics and Technology, Rzeszow University of Technology, 37-450 Stalowa Wola, Poland; m.bulakh@prz.edu.pl

**Keywords:** roughness, cold plasma, working surface, center plate, railway car

## Abstract

This paper presents a study on the use of cold plasma to reduce the roughness of the working surface of center plates of railway cars. The use of cold plasma is a promising method of surface treatment which allows for a significant reduction in roughness without changing the mechanical and chemical properties of the material. As part of the study, experiments were conducted on the treatment of center plates with cold plasma, the surface roughness was measured before and after the treatment, and the microhardness, microstructure, and chemical composition of the material were analyzed. The results of our experimental studies show that the use of cold plasma can reduce roughness by 1.4–1.6 times. At the same time, the roughness parameters $R_{a}$ are reduced by 29.1–37.4%, and $R_{z}$ by 29.3–39.6%. A slight increase of 2.81–3.31% in the roughness parameter $S_{a}$ is also obtained after cold plasma treatment compared to the base samples. Thus, the use of cold plasma for the treatment of center plates of railway cars can significantly increase their durability and reduce the costs of manufacture or repair, making this method promising for use in the railway industry.

## 1. Introduction

Ensuring the reliability and durability of vehicles has always been and remains one of the key tasks for the mechanical engineering and railway industries [1,2,3]. Under conditions of intensive use of transport infrastructure, railway cars are subject to significant operational loads, leading to accelerated wear of individual structural elements, such as center plates and center pads [4,5,6]. The center plate with the center pad of a railway car is one of the most important units, which serves to transfer vertical and horizontal loads between the car and the bogie and also ensures their stable adhesion and uniform distribution of the weight load [6,7].

Due to a high mechanical load and constant friction, center plates are subject to rapid wear, which is manifested in an increase in the roughness of their working surface [7]. Increased roughness leads to a number of negative consequences: the friction coefficient increases, negatively affecting the smoothness of the car and its controllability, and accelerated wear of the mating parts occurs, reducing the overall reliability of the structure and increasing the costs of maintenance and repair. When manufacturing and repairing center plates, grinding and polishing are used during final mechanical processing and are aimed at reducing the roughness of their surface [2,4]. However, these methods have a number of significant drawbacks. Firstly, they require significant labor costs and material resources. Secondly, such mechanical processing methods can lead to a change in the physical and mechanical properties of the material, which reduces its performance characteristics. Finally, the grinding process itself is quite energy-intensive and entails additional time spent on repair work.

Under conditions of increased competition and with the desire to improve the economic efficiency of railway transport production, the introduction of innovative technologies aimed at increasing the service life of car structural elements while reducing the costs of their maintenance and repair is becoming especially relevant [3,6]. One of the promising areas is the use of cold plasma for processing the working surfaces of railway car parts.

Cold plasma processing technology [8,9,10] is an advanced method that improves the surface characteristics of materials. This method involves the action of low-temperature plasma on the metal surface, an approach which can lead to improved performance characteristics [10,11,12]. It is important to note that cold plasma affects the material without significant heating, avoiding overheating and deformation of the material, such a phenomenon being a problem when using other processing methods.

The review of the current state and development directions of plasma arc surfacing technology for surface modification, presented in [13,14,15], is focused on improving wear resistance, corrosion, and erosion. These studies include various aspects of the process, in particular surfacing materials, process parameters, and process modifications, to improve the functional properties of coatings. A comparative analysis of surface plasma treatment is presented in [14]. However, the disadvantage of this work is the limitation of the scope of therapy.

The work in [15] evaluates the efficiency of cleaning the flat surfaces of selected materials using plasma. It shows the efficiency of cleaning surfaces under industrial or production conditions. However, the authors focus only on surface cleaning and do not provide other results.

According to the work in [16], the use of atmospheric plasma allows to significantly reduce the coefficient of friction and extend the service life of the tool. However, this work is limited to the study of the surface of cold rolling mill rolls only. In addition, the results of surface roughness measurements are not presented.

In the work presented in [17], the potential of cold plasma technology for the preparation of new materials for energy systems is demonstrated. Particular attention is paid to plasma-deposited optoelectronically active thin films for solar cells, nanocatalytic thin films for fuel cells, and plasma-treated carbon electrodes for supercapacitors. Although these results are important, they do not concern the treatment of alloyed steels or alloys and are not of interest to this study.

The study in [18] discusses the various applications of different cold plasma discharge mechanisms, plasma systems, and their interfacial adhesion characteristics in fiber-reinforced composites. The effects of different plasma sources and different discharge methods with modification procedures and their mechanisms on the changes in the structure and characteristics of high-performance fiber-reinforced composites are elucidated. The factors associated with cold plasma treatment applied to the surface modification of high-performance fibers are also analyzed. Despite the results presented, there is no information on the application of cold plasma treatment to alloy steels or alloys, limiting the relevance of the presented study to this paper.

The review in [19] discusses recent advances in the use of cold plasma for the fabrication of carbon-, metal-oxide-, and metal-nanoparticle-based materials and for modifying the surface properties of carbon-, metal-oxide-, and metal-nanoparticle-based materials. Future research opportunities and challenges are also suggested. The review focuses on cold plasma processing and modification of materials for rocket fuels, explosives, and pyrotechnics. In terms of relevance to this study, the review in [19] does not cover metallic materials.

An analysis of the literature shows that the use of plasma technologies in transport has significant potential. First of all, it can affect the increase in the efficiency, reliability, and quality of rolling stock repair, and can reduce the wear rate, which will lead to a decrease in the cost of frequent replacement of parts and to an increase in the productivity of repair enterprises. Due to the high accuracy and efficiency of plasma processes, enterprises can reduce the consumption of energy and materials during the processing of parts, a step which will reduce the overall operating costs for the repair and manufacture of rolling stock parts. In addition, plasma technologies are environmentally friendly, since they do not require the use of aggressive chemical solutions used in other grinding, polishing, and cutting methods. This reduces the harmful impact on the environment and on the working environment at enterprises.

As a result of the analysis, it was found that there are no research data on the use of cold plasma in the literature. In this regard, this study on the use of cold plasma to reduce the roughness of the working surface of a railway car center plate is relevant.

The relevance of this study is that the use of cold plasma for processing the surfaces of railway car center plates can be an important step to improve their performance characteristics. The use of this method can not only extend the service life of new center plates, but also reduce the frequency and cost of their repair. In the context of constant growth of transportation volumes and increasing requirements for reliability and safety of railway transport, increasing the service life of individual units and assemblies is becoming especially important.

To reduce the surface roughness of the central plate of a railway wagon, only mechanical finishing methods are used. However, the use of cold plasma treatment can substantially reduce labor costs compared to mechanical processing methods.

In addition, it is necessary to take into account that the railway industry strives to reduce costs for production and repair of rolling stock. The use of new technologies for processing materials, such as cold plasma, can improve the performance characteristics of units and reduce the costs of repair and restoration work. In the context of the need to improve economic efficiency and reduce operating costs, the use of advanced processing methods is becoming an important task for manufacturers and operators of railway transport.

Thus, the study of the effect of cold plasma on reducing the surface roughness of a railway car center plate not only meets the needs of modern industry for innovative technologies, but also helps solve practical problems associated with the increase in the reliability and durability of railway cars. These results can find wide application in the railway industry and can also be adapted for use in other areas where it is necessary to improve the surface characteristics of materials without changing their structure and physical properties.

The aim of this work is to study the use of cold plasma to reduce the roughness of the working surface of a railway car center plate. The study is expected to examine how effectively this method can be used to improve the performance characteristics of center plates, as well as how cold plasma treatment affects the mechanical and chemical properties of the material.

As a result of the study, we plan to identify the degree of influence of cold plasma on the reduction of surface roughness of the center plate and confirm the feasibility of using this method in industry settings. It is expected that the use of cold plasma will not only reduce the surface roughness of the material, but also maintain or improve the mechanical properties of the material, something which will significantly extend the service life of center plates and improve their performance characteristics. This opens up prospects for introducing the method into the repair and manufacturing processes of railway cars which, in turn, will increase the economic efficiency of production by reducing grinding costs.

## 2. Materials and Methods

### 2.1. Sample Preparation

Railway car center plates are made of alloy steel (the steel grade is classified by the manufacturer), the chemical composition of which is given in Table 1.

An important characteristic of the material is its mechanical properties, such as yield strength, tensile strength, relative elongation at break, and others. The mechanical properties of the sample material are given in Table 2.

The material of the prepared samples fully corresponded to the material of the center plate of the railway car. The samples were prepared as follows. The test surface was 25 × 12 mm in size, the length of the cut samples was 12 mm. The number of prepared samples was 30 units. Metal cutting was performed using a BP95d electrical discharge cutting machine (ZAP B.P., Kutnia, Poland) in order to minimize changes to the structure of the material.

Each sample was assigned a number to facilitate the tracking of the results during the experiment. The marking was made in such a way that it did not affect the sample itself and its properties (inscription on the non-working surface). All samples were subjected to the same preparation and processed under the same conditions. This ensured that comparable and accurate research data were obtained. The environmental conditions did not change. The ambient temperature was 20 °C, and the humidity was 5%. These two indicators had constant values. The samples were cleaned of dirt, oxides, and other impurities using chemical cleaning. The following solution was used to remove dirt and oxides: HCl—10–15% by volume, urotropine—0.1–0.3% by volume, and water—up to 84.7–89.1% of the solution volume. The samples were kept in the solution for 10–20 min, depending on the thickness of the oxide layers. After processing, they were thoroughly rinsed with water. The following solution was used for degreasing: NaOH—5–10% by weight, Na_3_PO_4_—3–5% by weight, and distilled water—up to 92%. The holding time was 15–30 min. After processing, they were rinsed with water.

That is, grinding and polishing were not performed.

### 2.2. Equipment for Processing the Test Surface of the Prepared Samples

**Figure 1:**
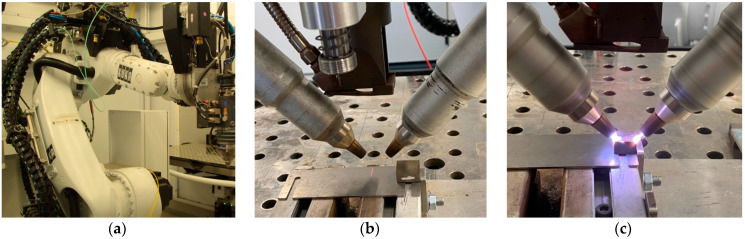
TruLaser Robot 5020 (**a**); (**b**,**c**) process of cold plasma treatment of the test surface of the prepared samples.

A TruLaser Robot 5020 (TRUMPF, Ditzingen, Germany) was used to process the test surface of the prepared samples (Figure 1a). This piece of equipment is equipped with a six-axis KUKA KR 30 HA robot (TRUMPF, Ditzingen, Germany), a tilt-and-turn controller with a maximum load of 400 kg, a rotary table with a maximum load of 250 kg per side, and a TruDisk4002 (TRUMPF, Ditzingen, Germany) laser source with a power of 4000 W with three heads. The process of cold plasma processing of the surface of the prepared samples is shown in Figure 1b,c.

The modes of cold plasma treatment applied to the test surface of the prepared samples on the TruLaser Robot 5020 are presented in Table 3.

The cold plasma treatment modes presented in Table 3 were selected through preliminary experimental studies.

### 2.3. Equipment and Methods for Measuring the Roughness of the Test Surface of Samples

Measuring the roughness of the studied samples’ surface is an important step in studying the microgeometry of the surface of materials, especially when the effect of treatment on the surface properties is investigated. In this case, the test surface of the samples was treated with cold plasma. Measuring the roughness allows us to determine how smooth or uneven the surface has become after treatment.

In this work, the contact roughness measurement method was used in accordance with ISO 21920-2:2021 [20].

Equipment for measuring surface roughness plays an important role in accurately assessing the change in the microgeometry of the test surface of the samples before and after treatment. Measurement of the roughness of the test surface of the prepared samples, both before and after cold plasma treatment, was performed on Hommel-Etamic T8000RC surface geometry testing equipment (HOMMEL-Etamic, Jena, Schwenningen, Germany).

The test surface of the samples was prepared, i.e., cleaned of dirt, dust, and other foreign substances that could distort the measurement results. In the study, the surface of the samples was not subjected to additional grinding or polishing after cutting in order to preserve the natural roughness. Cleaned samples were placed on the platform of the Hommel-Etamic T8000RC equipment for measurements.

A specific area for roughness measurements was allocated to each sample. In this study, the measurement area was 10 × 10 mm. This is a standard area sufficient to obtain representative data on the surface condition.

Measurements were carried out perpendicular to the lines of microprotrusions and irregularities, allowing for the most accurate recording of roughness over the entire area of the sample.

Measurements on the profilometer were carried out on a selected area with a step of 0.005 mm, which allowed for detailed recording of the slightest changes in the height of irregularities. The profilometer uses a scanning stylus that moves along the surface and records its topography. The number of repeated measurements on one sample was three. The result with the largest parameters $R_{a}$, $R_{z}$, and $S_{a}$ was then accepted. This allowed us to obtain detailed data on the microroughness of the studied sample surface, something which is especially important when studying changes after cold plasma treatment. Other roughness parameters were also recorded, but they are not presented in this paper.

### 2.4. Equipment and Methods for Studying the Microhardness of the Test Surface of the Prepared Samples

The study of the microhardness of the test surface of the prepared samples is of the utmost importance for assessing the effect of the cold plasma treatment on the mechanical properties of the material surface. The preservation or improvement of microhardness after the treatment may indicate that the method does not have a negative effect on the strength characteristics of the alloy steel, but it can contribute to the improvement of its operational properties.

The study of the microhardness of the test surface of the prepared samples was carried out in accordance with the ISO 6507-4:2018 [21] standard. The HV0.3 method was used, in which the load on the indenter was 2.94 N. In this case, the diameters of the diagonals of the indent print were measured.

The test surface of the samples was thoroughly cleaned and prepared to exclude the influence of contaminants on the measurement results.

To study the microhardness of the test surface of the prepared samples, a tester from ATM, brand QNESS 10/60 M (ATM Qness GmbH, Mammelzen, Germany), was used. This device is highly accurate. The device allows you to control the load on the indenter, ensuring accurate and stable results. To ensure the reliability of the microhardness data obtained using the HV0.3 method, five measurements were taken at different points on the surface of each sample. This is necessary to take into account possible variations in the properties of the material over the sample area. Based on the three obtained microhardness data points for the sample surface, the arithmetic mean value of microhardness was calculated. This gives an idea of the average level of microhardness of the sample surface and excludes random deviations. The average microhardness values were used for a comparative analysis of the state of the studied sample surface before and after cold plasma treatment.

### 2.5. Technique for Studying the Microstructure and Chemical Analysis of the Test Surface of the Material of the Prepared Samples

The study of the surface microstructure of the samples is aimed at identifying changes in the structure of the material after cold plasma treatment.

To study the microstructure and chemical analysis of the material of the prepared samples, a scanning electron microscope MIRA3 from TESCAN (Brno, Czechia) was used, making it possible to obtain highly detailed images of the surface microstructure of the material of the prepared samples.

The study used several magnification modes for a detailed analysis of the microstructure. At low magnification (×250), it was possible to get a general idea of the surface structure and identify large defects or macrostructural changes, while, at higher magnifications (×1000–×10,000), it was possible to study microroughness, texture, and other small elements.

MIRA3 allows you to record both topography and contrast associated with differences in the chemical composition and density of the material, something which is especially important when studying heterogeneous materials. All obtained microstructure images were recorded for further analysis. The MIRA3 microscope software (Essence™) allows the measurement of the sizes of the detected structural elements and their comparison among samples before and after treatment. These data serve as the basis for a qualitative and quantitative assessment of microstructure changes under the influence of cold plasma.

Before studying the microstructure, the samples were carefully prepared: they were cleaned of dirt, oxides, and other impurities that could distort the results. In the case of this study, the surface of the samples was not subjected to additional grinding or polishing after cutting, since the goal was to study the effect of cold plasma on the initial surface roughness of samples treated with cold plasma.

A specific area (site) was selected on each sample for detailed analysis. This was done in order to perform measurements on the most typical areas of the test surface of the samples. The study area was representative of the entire test surface (10 × 10 mm) to exclude the influence of local defects.

To analyze the chemical composition, electron images of the microstructure of the samples after cold plasma treatment were selected. In this case, several areas on the surface were selected for spectral analysis so that the results were representative and covered different treatment zones. The analysis was performed at different points of the samples to obtain a complete picture of the distribution of chemical elements on the surface.

During the chemical analysis of the test surface of the prepared samples after cold plasma treatment, electron images of the microstructure were selected, and the chemical composition was recorded in the automatic software mode (Oxford Instruments, Abingdon, UK).

## 3. Results and Discussion

### 3.1. Roughness of the Studied Sample Surface

#### 3.1.1. Roughness of the Test Surface of the Base Samples

**Figure 2:**
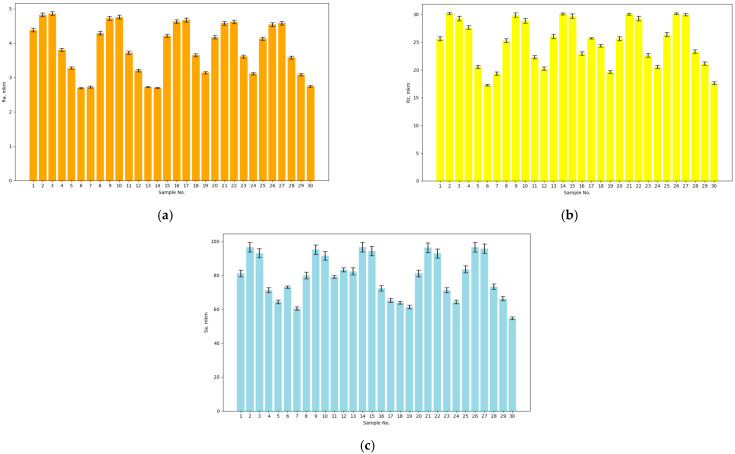
Results from the measurement of the surface roughness parameters of the test samples before treatment: (**a**) $R_{a}$, (**b**) $R_{z}$, (**c**) $S_{a}$.

The results of measuring the surface roughness parameters of the test samples before treatment are shown in Figure 2.

**Figure 3:**
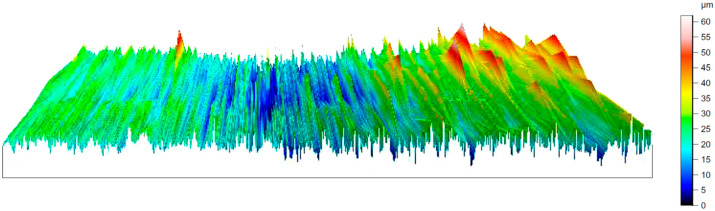
Three-dimensional model of the roughness of the test surface of the basic prepared samples.

The measurements and the presented results (Figure 2) show that the values of the roughness parameter $R_{a}$ for the test surface of the base samples were in the range of 2.680–4.920 μm. The values of the roughness parameter $R_{z}$ for the test surface of the base samples were in the range of 17.100–30.300 μm. The values of the roughness parameter $S_{a}$ were in the range of 53.790–99.910 µm. As a result of the roughness measurements of the prepared base samples, a 3D model was built. The result is shown in Figure 3.

#### 3.1.2. Roughness of the Test Surface of the Samples After Cold Plasma Treatment

**Figure 4:**
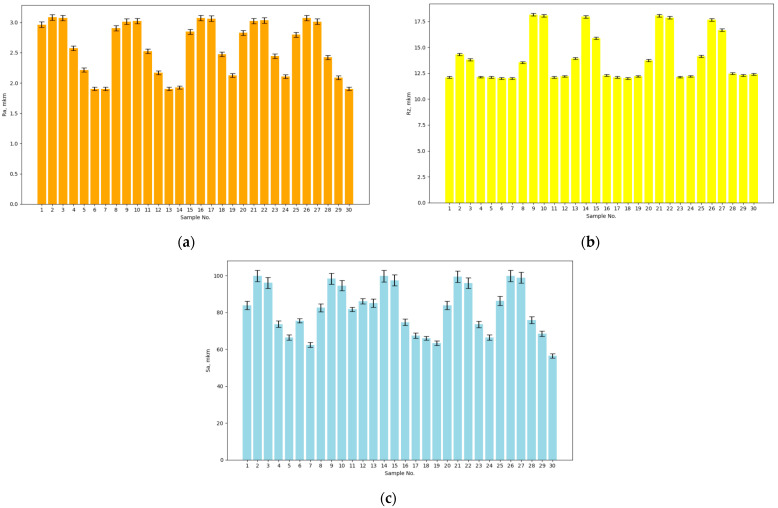
Results from the measurement of the roughness parameters of the test surface of the prepared samples after cold plasma treatment: (**a**) $R_{a}$, (**b**) $R_{z}$, (**c**) $S_{a}$.

The roughness measurements of the test surface of the prepared samples after cold plasma treatment are shown in Figure 4.

The performed roughness measurements and the results presented in Figure 4 show that the values of the roughness parameter $R_{a}$ for the test surface of the prepared samples after cold plasma treatment were in the range of 1.900–3.080 μm. As a result, we obtained a decrease in the roughness parameter $R_{a}$ after cold plasma treatment by 29.1–37.4% compared to the base samples.

The values of the roughness parameter $R_{z}$ for the test surface of the prepared samples after cold plasma treatment were in the range of 12.100–18.300 μm. That is, after cold plasma treatment of the test surface of the prepared samples, the value of the roughness parameter $R_{z}$ decreased by 29.3–39.6% compared to the base samples.

The values of the roughness parameter $S_{a}$ for the test surface of the prepared samples after cold plasma treatment were in the range of 55.300–103.210 μm. As a result, a slight increase in the roughness parameter $S_{a}$ after cold plasma treatment was obtained by 2.81–3.31% compared to the base samples.

**Figure 5:**
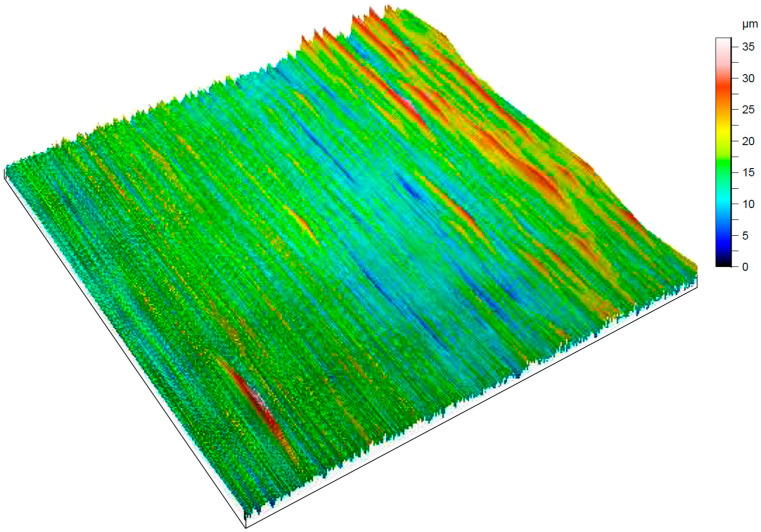
Three-dimensional model of the roughness of the test surface of the prepared samples after cold plasma treatment.

Alongside the obtained roughness measurements of the test surface of the prepared samples after cold plasma treatment, a 3D model was also built. The result is shown in Figure 5.

The three-dimensional models (Figure 3 and Figure 5) exhibited an identical roughness topology. However, the values of the protrusions and depressions of the test surface of the prepared samples after cold plasma treatment (Figure 5) were 1.4–1.6 times lower compared to the roughness of the test surface of the prepared base samples. This confirms the feasibility of using cold plasma to reduce the roughness of the test surface of the prepared samples.

To confirm the feasibility of using cold plasma, it was necessary to study the physical, mechanical, and physicochemical properties of the sample material. This is due to the fact that the effect of cold plasma on the surface of the prepared samples can lead to a decrease in the physical, mechanical, and physicochemical properties of the material. Therefore, the results of studies on the microhardness and chemical composition of the test surface of the prepared samples are presented below.

### 3.2. Microhardness Test Results

During the study of the microhardness of the test surface of the prepared samples, both before and after the cold plasma treatment, it was found that the microhardness value was in the range of 537–645 HV.

The number of measurement points was taken as five to obtain more accurate data on the average value of the microhardness of the test surface.

**Figure 6:**
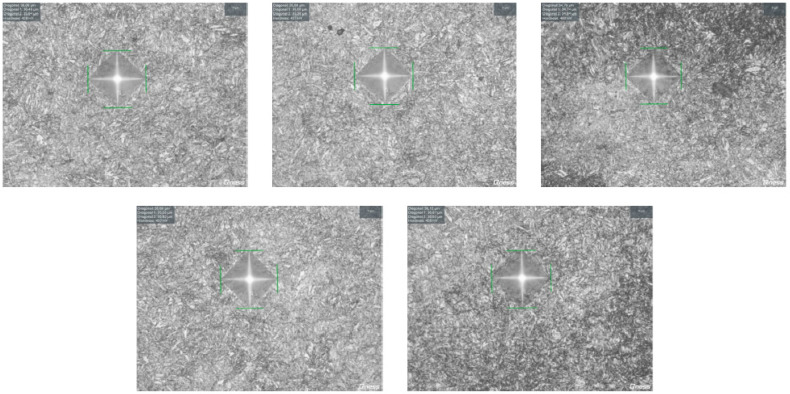
Test point images of the test surface of the prepared sample (sample No. 4) after cold plasma treatment.

Test point images of the test surface of the prepared samples after the cold plasma treatment are shown in Figure 6.

Figure 6 shows the results in the form of images for sample No. 4 with pyramid indentation. Microhardness was calculated based on the diagonal sizes d_1_ and d_2_. The images show that the pyramid indentation depth had the same tendency. The diagonal sizes were the same and were equal to d_1_ = d_2_ = 0.04 mm. The data provided indicate a uniform distribution of microhardness across the surface depth of the samples under study.

**Figure 7:**
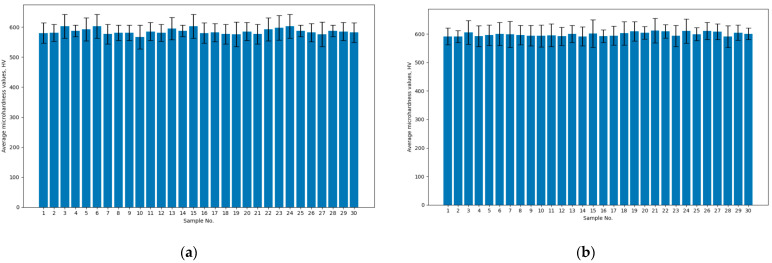
Average values of microhardness of the test surface of the prepared samples before (**a**) and after (**b**) cold plasma treatment.

The average values of microhardness of the test surface of the prepared samples before and after the cold plasma treatment are shown in Figure 7.

From the presented results (Figure 7a), it follows that the average values of microhardness of the test surface of the prepared samples before the treatment differed by 6.01%. The average values of microhardness of the test surface of the prepared samples after the cold plasma treatment (Figure 7b) differed by 3.46%. The difference between the average values of microhardness of the test surface of the prepared samples before and after the treatment was estimated at 1.50–4.09%.

The presented results from the study of the microhardness of the test surface of the prepared samples after the cold plasma treatment confirm that the mechanical properties of the sample material did not change. This confirms the feasibility of using cold plasma to reduce the roughness of the test surface of the prepared samples.

### 3.3. Results of the Study of the Microstructure of the Test Surface of the Prepared Samples Before and After the Cold Plasma Treatment

**Figure 8:**
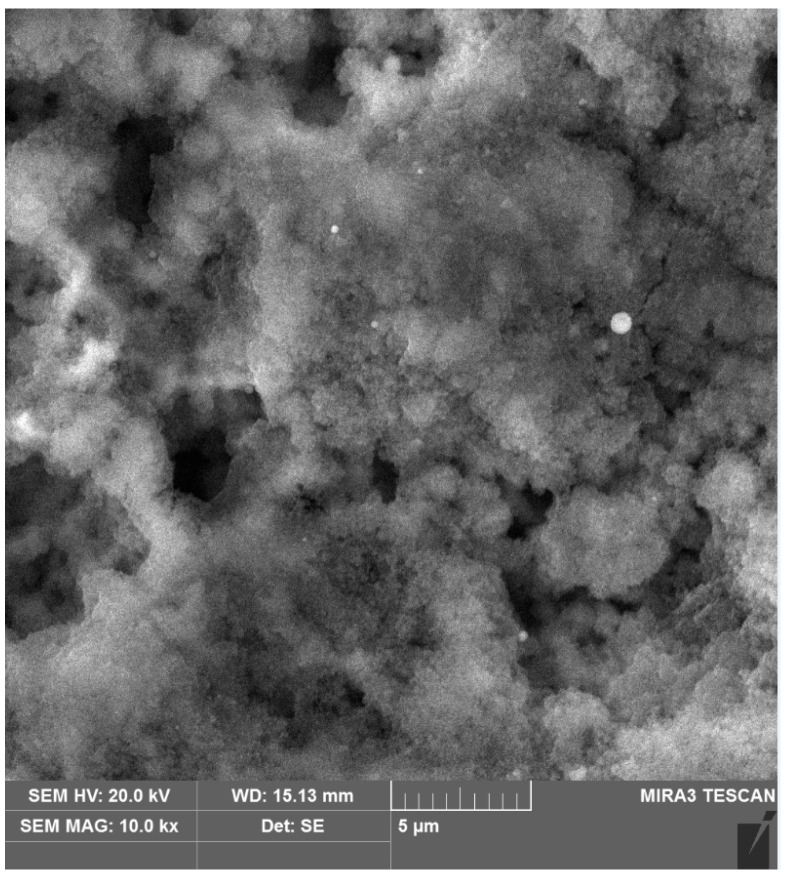
Microstructure of the test surface of the prepared sample before the cold plasma treatment, ×10,000.

The microstructure of the test surface of the prepared sample before the cold plasma treatment is shown in Figure 8.

**Figure 9:**
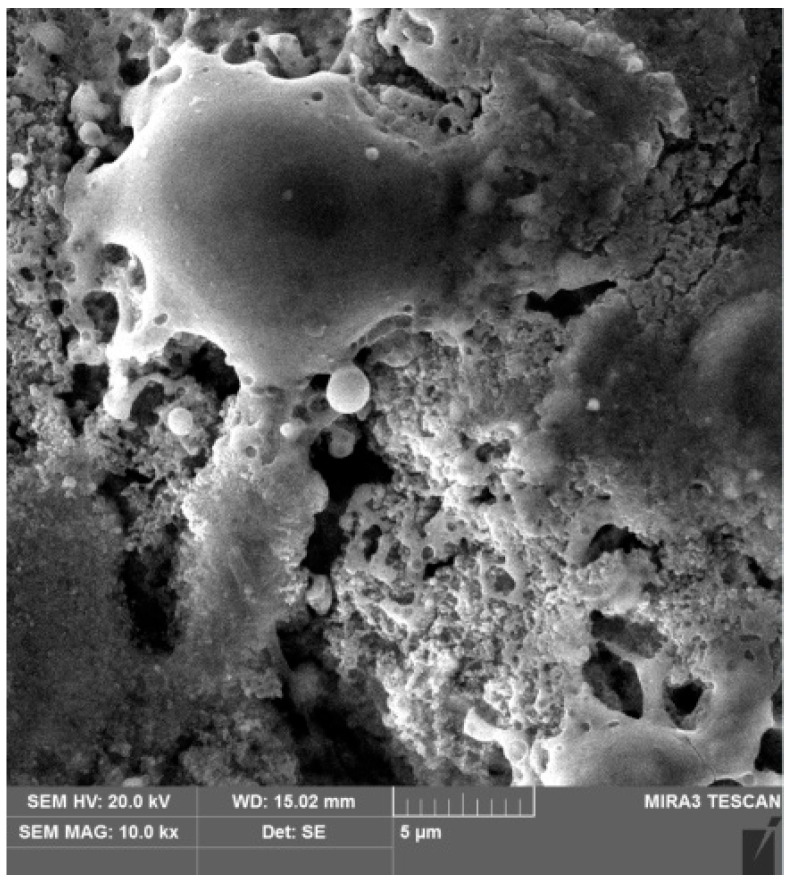
Microstructure of the test surface of the prepared sample after the cold plasma treatment, ×10,000.

The microstructure of the test surface of the prepared sample after the cold plasma treatment is shown in Figure 9.

The obtained images of the microstructure of the test surface of the prepared samples before (Figure 8) and after the cold plasma treatment (Figure 9) can be distinguished by the presence of melted islands. There were no noticeable changes in the microstructure of the test surface of the prepared samples before treatment (Figure 8) and after the cold plasma treatment (Figure 9). At the same time, the image (Figure 9) demonstrates a smoothing of the surface which is consistent with the results of roughness measurements. In Figure 8, the melted islands after the cold plasma treatment are clearly visible.

This confirms the decrease in roughness, the results of which were obtained earlier. The effect of cold plasma on the protrusions of the roughness consists of the melting of these protrusions, leading to a decrease in the roughness parameters $R_{a}$ and $R_{z}$. It is important to note that the structure of the material in the deep layers of the prepared samples remained unchanged after the cold plasma treatment, indicating minimal impact of the plasma on the material itself and preservation of its basic properties. The use of the MIRA3 scanning electron microscope in this study made it possible to clearly demonstrate how cold plasma affects the microroughness of the alloy steel surface. This is confirmed by visual changes in the microphotographs, which show alignment and smoothing of the protrusions which, in turn, explain the reduction in roughness.

### 3.4. Results of the Study of the Chemical Composition of the Surface of the Prepared Samples Before and After the Cold Plasma Treatment

During the chemical analysis of the surface of the prepared samples before and after the cold plasma treatment, the chemical composition of this material was obtained. The results are presented in Table 4 and Table 5. The software (Essence™) recorded the spectra of the chemical composition for each selected area on the test surface of the prepared samples after the cold plasma treatment. Based on the obtained data, tables were constructed indicating the percentage content of each element found on the surface. Particular attention was paid to whether the chemical composition of the test surface of the prepared samples changed after exposure to cold plasma. For this purpose, the data on the base samples were compared with the results obtained after the treatment to find out whether the plasma caused changes to the content of elements on the surface.

From Table 4 and Table 5, it can be seen that the chemical composition of the test surface of the prepared samples before and after the cold plasma treatment differed slightly. The discrepancy in the percentage composition of the material of the base samples and samples after the cold plasma treatment did not exceed 2.5%. Energy dispersive analysis has a high accuracy, but small discrepancies in the percentage content of the elements are possible due to the error of the method. It is important to take into account that differences in the chemical composition within 2.5% before and after treatment can be considered insignificant, as they can be due to statistical fluctuations. The difference between the average values of the percentage chemical composition of the test surface of the prepared samples before and after the cold plasma treatment did not exceed 1.0%.

The presented results indicate that the action of the cold plasma did not affect the chemical composition of the material. This is an important point that cold plasma does not change the chemical composition of the material, a point which should be taken into account in further research. Almost every known method which is intended for processing in order to improve the physical and mechanical properties of surfaces makes changes to the chemical composition of the surface of materials. The stability of the chemical composition after the cold plasma treatment is an important factor for the use of this method in industry settings. Preservation of the original chemical characteristics of the material ensures that the treatment will not lead to undesirable changes in the properties of alloy steel, such as corrosion resistance or mechanical strength. These data are especially important for applications in those industries where it is critical to maintain the consistency of the chemical composition while improving the surface characteristics of the material.

## 4. Conclusions

This paper presents a study on the use of cold plasma to reduce the roughness of the working surface of a railway car center plate. In general, this paper presents, for the first time, the results of a study on the use of cold plasma treatment on alloy steel surfaces. The results of these studies confirm the feasibility of using cold plasma to treat alloy steel surfaces and are as follows:

1. The roughness index $R_{a}$ for the treated samples decreased by 29.1–37.4%, while the index $R_{z}$ decreased by 29.3–39.6% compared to the initial base values. A slight increase of 2.81–3.31% in the roughness parameter $S_{a}$ was also obtained after the cold plasma treatment compared to the base samples. The measurements presented in the form of graphs and 3D models demonstrate that the cold plasma treatment significantly changed the surface topology, reducing the height of protrusions and depressions by 1.4–1.6 times.
2. It was found that the microhardness of the prepared samples changed insignificantly and remained practically at the same level as before the treatment. The difference between the average values of the microhardness of the test surface of the prepared samples before and after the treatment was estimated at 1.50–4.09%.
3. The study of the microstructure of the samples after the cold plasma treatment revealed the following effects. In the microphotographs taken with a magnification of 250 to 10,000 times, melted islands were observed on the surface, indicating the effect of the cold plasma on the protrusions of microroughness. This process resulted in the melting of surface protrusions which, in turn, reduced roughness.
4. The analysis of the chemical composition of the test surface of the samples after the cold plasma treatment showed minimal changes in the content of different elements. The percentage deviation in the composition did not exceed 1.0%, which is within the measurement error and indicates that cold plasma did not elicit significant changes to the chemical composition of the material. This fact is of particular value, since most methods aimed at improving the surface properties of materials, to one degree or another, change the chemical composition of said material, possibly leading to a change in the properties of the material as a whole.

The presented research results confirm the effectiveness of using cold plasma to reduce the surface roughness of alloy steel, opening up opportunities to improve the performance of the center plate of a railway car. These results also indicate the promise of this method for industrial use, especially in those industries where surface smoothness and minimization of microroughness are critical. In the future, the possibility of using this method not only for alloy steels but also for other materials where high surface smoothness and preservation of the original mechanical characteristics are required should be considered. Also, in the future, we plan to perform a comparative assessment of labor costs and economic efficiency of applying the cold plasma treatment to the surfaces of different railway car parts.

## Figures and Tables

**Table 1:** Chemical composition of the material of railway car center plates.

Element	Si	Cr	C	Mn	Ni	Cu	Zn	Fe
%	0.40	1.00	0.40	0.80	1.45	6.60	4.35	85.00

**Table 2:** Mechanical properties of the material of railway car center plates.

Parameter	Sample Size, mm	Test Temperature, °C	Yield Strength, σ_0.2_, MPa	Tensile Strength, σ_b_, MPa	Elongation at Break, δ, %	Relative Narrowing, ψ, %	Impact Strength KCU, kJ/m^2^	Hardness, HRC
Meaning	25 × 12 × 100	20	990	1580	26	44	109	52.4

**Table 3:** The modes of cold plasma treatment of the test surface of prepared samples on the TruLaser Robot 5020.

Modes	Voltage Range, V	Current Strength, A	Pressure, mbar	Number of Repetitions	Rotation Speed, rpm	Power, W
Values	316–318	4.1–4.3	28–29	3	2840–2890	330–342

**Table 4:** Results of the chemical analysis of the surface of the samples before the treatment.

Element	Si	Cr	C	Mn	Ni	Cu	Zn	Fe
Maximum value, %	0.40	1.01	0.41	0.81	1.46	6.64	4.38	85.11
Minimum value, %	0.39	0.99	0.40	0.79	1.43	6.55	4.28	84.32
Mean value, %	0.398	1.001	0.404	0.796	1.453	6.597	4.345	84.993
Deviation, σ, %	0.054	0.061	0.019	0.072	0.038	0.257	0.192	0.641

**Table 5:** Results of the chemical analysis of the surface of the samples after the cold plasma treatment.

Element	Si	Cr	C	Mn	Ni	Cu	Zn	Fe
Maximum value, %	0.41	1.00	0.41	0.82	1.47	6.65	4.37	85.07
Minimum value, %	0.40	0.99	0.4	0.8	1.45	6.57	4.29	84.35
Mean value, %	0.402	0.997	0.406	0.813	1.464	6.611	4.338	84.892
Deviation, σ, %	0.011	0.016	0.033	0.071	0.033	0.213	0.263	0.975

## Data Availability

The original contributions presented in the study are included in the article, further inquiries can be directed to the corresponding author: m.bulakh@prz.edu.pl.

## References

[1] Baranovskyi D., Myamlin S. (2023). The criterion of development of processes of the self organization of subsystems of the second level in tribosystems of diesel engine. Sci. Rep..

[2] Baranovskyi D., Myamlin S., Kebal I. (2022). Increasing the Carrying Capacity of the Solid-Body Rail Freight Car. Adv. Sci. Technol. Res. J..

[3] Panchenko S., Gerlici J., Lovska A., Ravlyuk V., Dižo J., Blatnický M. (2024). Analysis of Asymmetric Wear of Brake Pads on Freight Wagons despite Full Contact between Pad Surface and Wheel. Symmetry.

[4] Chmielowiec A. (2021). Algorithm for error-free determination of the variance of all contiguous subsequences and fixed-length contiguous subsequences for a sequence of industrial measurement data. Comput. Stat..

[5] Gerlici J., Lovska A., Vatulia G., Pavliuchenkov M., Kravchenko O., Solčanský S. (2023). Situational Adaptation of the Open Wagon Body to Container Transportation. Appl. Sci..

[6] Fomin O.V., Lovska A.O., Fomina A.M., Turpak S.M., Hrytsai S.V. (2022). Load of the wagon-platform for transportation of bulk cargoes. Sci. Bull. Natl. Min. Univ..

[7] Baranovskyi D., Myamlin S., Podosonov D., Muradian L. (2023). Determination of the filler concentration of the composite material to reduce the wear of the central bowl of the rail truck bolster. Ain Shams Eng. J..

[8] Łatka L., Biskup P. (2020). Development in PTA surface modifications—A review. Adv. Mater. Sci..

[9] Bhat K.U., Panemangalore D.B., Kuruveri S.B., John M., Menezes P.L. (2022). Surface modification of 6xxx Series aluminum alloys. Coatings.

[10] Yuan S., Lin N., Zeng Q., Zhang H., Liu X., Wang Z., Wu Y. (2020). Recent developments in research of double glow plasma surface alloying technology: A brief review. J. Mater. Res. Technol..

[11] Pesode P., Barve S. (2021). Surface modification of titanium and titanium alloy by plasma electrolytic oxidation process for biomedical applications: A review. Mater. Today Proc..

[12] Zhang L.C., Chen L.Y., Wang L. (2020). Surface modification of titanium and titanium alloys: Technologies, developments, and future interests. Adv. Eng. Mater..

[13] Rakhadilov B.K., Kenesbekov A.B., Kowalevski P., Ocheredko Y.A., Sagdoldina Z.B. (2020). Development of air-plasma technology for hardening cutting tools by applying wear-resistant coatings. News Natl. Acad. Sci. Repub. Kazakhstan Ser. Geol. Tech. Sci..

[14] Jaffer Z.J., Abdalameer N.K., Noori A.S. (2024). Plasma Surface Treatment of Metals: A Comprehensive Review of Recent Developments and Future Prospects. Int. J. Nanosci..

[15] Janík R., Vargová V., Kohutiar M., Krbata M., Moricová K. (2024). The capability of diffuse coplanar surface barrier plasma to effectively remove contaminants from surfaces of various materials. Materialwiss.

[16] Gonçalves J.L., De Mello J.D.B., Costa H.L. (2021). Tribological behaviour of alternative surface modifications for cold rolling mill rolls. Wear.

[17] Tyczkowski J. (2011). New materials for innovative energy systems produced by cold plasma technique. Funct. Mater. Lett..

[18] Wu M., Jia L., Lu S., Qin Z., Wei S., Yan R. (2021). Interfacial performance of high-performance fiber-reinforced composites improved by cold plasma treatment: A review. Surf. Interfaces.

[19] Men Y.-L., Liu P., Meng X.-Y., Pan Y.-X. (2022). Recent progresses in material fabrication and modification by cold plasma technique. FirePhysChem.

[20] (2021). Geometrical Product Specifications (GPS)—Surface Texture: Profile—Part 2: Terms, Definitions and Surface Texture Parameters.

[21] (2018). Metallic Materials—Vickers Hardness Test—Part 4: Tables of Hardness Values.

